# Two key polymorphisms in a newly discovered allele of the *Vitis vinifera TPS24* gene are responsible for the production of the rotundone precursor α-guaiene

**DOI:** 10.1093/jxb/erv491

**Published:** 2015-11-17

**Authors:** Damian Paul Drew, Trine Bundgaard Andersen, Crystal Sweetman, Birger Lindberg Møller, Christopher Ford, Henrik Toft Simonsen

**Affiliations:** ^1^Plant Biochemistry Laboratory, Copenhagen Plant Science Centre, Department of Plant and Environmental Sciences, Faculty of Science, University of Copenhagen, DK-1871 Frederiksberg C, Denmark; ^2^Wine Science, School of Agriculture, Food and Wine, University of Adelaide, Urrbrae SA 5064, Australia

**Keywords:** Pinot Noir, rotundone, sesquiterpene synthase, sesquiterpenoids, Shiraz, wine aroma.

## Abstract

Rotundone was initially identified as a grape-derived compound responsible for the peppery aroma of Shiraz wine varieties. It has subsequently been found in black and white pepper and several other spices. Because of its potent aroma, the molecular basis for rotundone formation is of particular relevance to grape and wine scientists and industry. We have identified and functionally characterized *in planta* a sesquiterpene synthase, VvGuaS, from developing grape berries, and have demonstrated that it produces the precursor of rotundone, α-guaiene, as its main product. The VvGuaS enzyme is a novel allele of the sesquiterpene synthase gene, *VvTPS24*, which has previously been reported to encode VvPNSeInt, an enzyme that produces a variety of selinene-type sesquiterpenes. This newly discovered *VvTPS24* allele encodes an enzyme 99.5% identical to VvPNSeInt, with the differences comprising just 6 out of the 561 amino acid residues. Molecular modelling of the enzymes revealed that two of these residues, T414 and V530, are located in the active site of VvGuaS within 4 Å of the binding-site of the substrate, farnesyl pyrophosphate. Mutation of these two residues of VvGuaS into the corresponding polymorphisms in VvPNSeInt results in a complete functional conversion of one enzyme into the other, while mutation of each residue individually produces an intermediate change in the product profile. We have therefore demonstrated that VvGuaS, an enzyme responsible for production of the rotundone precursor, α-guaiene, is encoded by a novel allele of the previously characterized grapevine gene *VvTPS24* and that two specific polymorphisms are responsible for functional differences between *VvTPS24* alleles.

## Highlight

VvGuaS, a novel allele of the *VvTPS24* gene, is responsible for the production of the rotundone precursor α-guaiene. Two specific polymorphisms distinguish VvGuaS from its non-guaiene-producing homologue VvPNSeInt.

## Introduction

Terpenoids are one of the most diverse and abundant classes of specialized metabolites in the plant kingdom and perform a variety of functions, including defence against insects and microbes (Pare and Tumlinson, 1999) or the attraction of pollinators (Martin *et al.*, 2009). Many terpenoids are volatile and therefore have the potential to act as aroma compounds, a function that is particularly relevant in the cultivated grapevine *Vitis vinifera* L., owing to the use of its berries in wine making (Lund and Bohlmann, 2006; Dunlevy *et al.*, 2009). There have been several studies into the effect of 10-carbon monoterpenoids, such as linalool, nerol, and geraniol, on the muscat aroma of white wine varieties (Sanchez-Palomo *et al.*, 2005; Vilanova and Sieiro, 2006; Dziadas and Jelen, 2010), and into the genetic basis for monoterpene biosynthesis in grapes (Martin and Bohlmann, 2004; Battilana *et al.*, 2011; Martin *et al.*, 2012). In recent studies, the presence of 15-carbon sesquiterpenoids in grapes has been investigated (Coelho *et al.*, 2006; Kalua and Boss, 2010; May and Wust, 2012; Matarese *et al.*, 2013; Matarese *et al.*, 2014), although their presence in wine and the individual and collective effect of sesquiterpenes on wine aroma are poorly understood (Robinson *et al.*, 2014b).

In 2007, the sesquiterpene ylangene was identified as a ‘marker compound’ for the peppery aroma in Shiraz wine, although the chemical itself was not found to have a significant aroma (Parker *et al.*, 2007). A different bicyclic sesquiterpene, rotundone, was subsequently identified as the compound responsible for the peppery aroma (Wood *et al.*, 2008). Rotundone exhibits extremely low detection thresholds of 8ng/L in water and 16ng/L in wine (Siebert *et al.*, 2008). This specialized plant metabolite is a stable sesquiterpenoid composed of a guaiene carbon skeleton with a single ketone group in the carbon 2 position. Its identification in Shiraz represented the first demonstration of a specific sesquiterpene directly linked to an aroma characteristic in wine. Given the potent effect of this single volatile metabolite, an understanding of the gene or genes responsible for rotundone biosynthesis in grapes will provide a platform to select for desired levels of rotundone in different grape varieties. This could include utilizing techniques such as marker-assisted selection or metabolic engineering for the purpose of producing wines with increased or less peppery character. Such options have previously been outlined with respect to grape monoterpene content following the discovery of a polymorphism within 1-deoxy-D-xylulose 5-phosphate synthase responsible for increased linalool, nerol, and geraniol content in berries of some varieties (Battilana *et al.*, 2011).

The precise mechanism of rotundone biosynthesis is unknown. Based on its structure, the direct precursor is likely to be α-guaiene, which can be formed in a single step from farnesyl pyrophosphate (FPP) by a terpene synthase (TPS) (Kumeta and Ito, 2010). This hypothesis is further supported by recent evidence that α-guaiene can be converted to rotundone via a non-specific oxidation reaction mediated by a fungal laccase enzyme in the presence of chemical mediators (Schilling *et al.*, 2013) or by direct oxidation (Huang *et al.*, 2014). Furthermore, rotundone is found in plants that also contain α-guaiene and other guaiene-type sesquiterpenes (bicyclic compounds comprising 5-carbon and 7-carbon rings), including pepper (*Piper* spp.; Jirovetz *et al.*, 2002), agarwood (*Aquilaria* spp.; Ishihara *et al.*, 1991), and some *Cyperus* spp. (Ishihara *et al.*, 1991; Olawore *et al.*, 2006; Kumeta and Ito, 2010). α-Guaiene has been identified in numerous plants and in essential oils (Pandey *et al.*, 2003; Wei and Shibamoto, 2007; Sundaresan *et al.*, 2009; Pripdeevech *et al.*, 2011) and has thus been more widely detected in comparison to rotundone. This may, however, be due to differences in concentration and ease of detection. Following the initial identification of rotundone in Shiraz grapes and wine, it has been found in a number of other red and white wine varieties, demonstrating that it is not unique to a single grapevine cultivar (Caputi *et al.*, 2011; Mattivi *et al.*, 2011; Matarese *et al.*, 2014; Scarlett *et al.*, 2014). Nevertheless, the absence of rotundone in many varieties could suggest that genes responsible for its biosynthesis, or for the biosynthesis of its precursor, may be absent or differentially expressed in some varieties.

In plants, TPSs are encoded by a multigene family with up to 150 members in some species (Chen *et al.*, 2011). The TPS family has been relatively well defined in grapevine, with 69 full-length *TPS* genes identified on the published genomic scaffold based on an inbred Pinot Noir genome, PN40024 (Jaillon *et al.*, 2007; Martin *et al.*, 2010). These included 30 potential genes of the *TPS-a* subfamily, generally considered responsible for sesquiterpene biosynthesis, which were found to exist in two clusters on chromosomes 18 and 19. Of these, 13 of the encoded enzymes were functionally characterized by Martin *et al.* (2010) using *Escherichia coli* engineered to produce and accumulate the substrate FPP. Other grape-derived TPSs have previously been functionally characterized in other studies (Lucker *et al.*, 2004; Martin *et al.*, 2009; Martin *et al.*, 2012; Matarese *et al.*, 2014). While there is no clear candidate for the gene responsible for the biosynthesis of a rotundone precursor, a recombinantly expressed protein from Pinot Noir named VvPNSeInt, whose main products were selinene and intermedeol, also produced α-guaiene as a minor product (3.5%) (Martin *et al.*, 2010).

In order to investigate the possibility that polymorphic differences in *TPS* genes could alter their function, we amplified a number of TPS cDNAs from developing grape berries of the Shiraz variety, a variety known to produce rotundone. Here we report the functional characterization and mutagenic analysis of one of the encoded enzymes by transient expression in *Nicotiana benthamiana* leaves. We demonstrate that a novel allele of *VvTPS24*, the gene expected to encode VvPNSeInt, actually encodes VvGuaS, an enzyme with predominantly α-guaiene synthase activity. Furthermore, we show that two specific nucleotide substitutions in the *VvTPS24* gene can change its encoded product from VvGuaS into VvPNSeInt, and thus relate two grapevine polymorphisms with a physiologically relevant functional outcome.

## Materials and Methods

### Plant material and nucleic acid isolation and amplification

Total RNA was isolated from *Vitis vinifera* cv. Shiraz grown in the Nuriootpa Research vineyard, Barossa Valley, South Australia, and harvested at various times of the 2013 season. Harvested grapes were frozen immediately in liquid nitrogen and stored at −80°C until required. RNA extractions were carried out as described (Sweetman *et al.*, 2012) and cDNA was synthesized using the iScript™ Select cDNA Synthesis Kit (Bio-Rad) as per the manufacturer’s instructions. *VvTPS24* was amplified from cDNA using Phusion® High-Fidelity DNA Polymerase (NEB) with the primer pair 5′- CACCATGTCTGTTCCACTATCAGTCTCAG-3′ and 5′-TTACATTGGCACAGGATCTATG-3′ under standard PCR conditions. In the first instance, amplicons were ligated into pENTR/D-TOPO (Invitrogen) and at least three clones were sequenced (AGRF, Adelaide).

### Site-directed mutagenesis

Mutations were carried out on cDNAs in the pENTR/D-TOPO vector using an adaptation of the QuikChange Site Directed Mutagenesis kit (Stratagene) essentially as described (Drew *et al.*, 2011). In short, the complete T414S mutant was amplified, complete with plasmid, using the mutagenesis primer 5′-GCAACGCGCTGGTAA**G**C TCTGCCTGCTCTATG-3′ and its reverse complement pair (non-complementary nucleotide shown in bold). The V530M mutant was made with the 5′-CTGAATTTTAGCCGGATG**A** TGGACGTCTTGTACAA-3′ primer and its reverse complement pair. The double mutant T414S/V530M was created in a two-step process by carrying out the V530M mutation using the T414S mutant as a template. All products were confirmed via sequencing.

### Homology modelling

The coordinates of 5-epi-aristolochene synthase (TEAS; Protein Data Bank accession 5EAT) (Starks *et al.*, 1997) were used as a template structure for homology modelling of the VvGuaS sequence. This template structure included a structural analogue of FPP bound within the active site for determination of its proximity to residues forming the surface of the binding cavity. Homology modelling was performed using satisfaction of spatial restraints of all non-hydrogen atoms using MODELLER version 7.2 (Eswar *et al.*, 2003). All figures were generated using PyMOL (DeLano Scientific LLC, San Francisco, CA, USA) and modelling of mutated amino acid residues was carried out within the VvGuaS model using the PyMOL residue mutation function.

### *Heterologous expression in* Nicotiana benthamiana *and solid-phase micro-extraction*

Forward and reverse primers were designed with USER-overhangs to enable cloning into a USER-compatible version of the pEAQ-*HT* vector. USER cloning was performed as previously described (Nour-Eldin *et al.*, 2006). The pEAQ-*HT* plasmid (kindly provided by George Lomonosonoff, John Innes Research Centre, Norwich, UK) harbouring the viral suppressor p19 was modified to harbour a USER cassette. *N. benthamiana* plants were grown from seeds at 24°C day/19°C night cycles for 5 weeks before infiltration. Transformation of *Agrobacterium tumefaciens* and subsequent expression in *N. benthamiana* were performed as described previously (Yang *et al.*, 2012; Bach *et al.*, 2014). A whole leaf from infiltrated *N. benthamiana* was inserted into a 20ml glass vial and the volatiles extracted at 60°C for 20min using a Supelco 57298-U, 50/30 μm divinylbenzene/carboxen/polydimethylsiloxane StableFlex/SS (1cm) fibre (Supelco Sigma-Aldrich, Denmark). Fibres were reconditioned for 20min at 240°C. Analysis was performed in triplicate for each sample and *N. benthamiana* leaves expressing p19 from the pEAQ-*HT* vector were used as controls for comparison.

### GC-MS method and data analysis

Samples were analysed on a Shimadzu GCMS‐QP2010 Plus using an Agilent HP-5ms Ultra Inert fused Silica capillary column of 29 m length × 0.25 mm diameter × 0.25 μm film thickness, inserted directly into the ion source of the MS (Simonsen *et al.*, 2009; Drew *et al.*, 2013). The pressure was kept at 16 kPa, giving a column flow of 1.25mL/min. The injection port temperature was set to 250°C, and lower injection port temperatures were investigated to confirm that a significant degree of thermal degradation did not occur (Andersen *et al.*, 2015). The oven temperature was set to 60°C for 3min, and increased to 160°C at a rate of 7°C/min, before a further increase to 300°C at a rate of 50°C/min. This temperature was held for 5min and finally increased to 320°C at a rate of 50°C/min, where it was maintained for 3min. The carrier gas was H_2_ and the ionization electron energy was 70eV. The ion source temperature was 230°C with an interface temperature of 280°C. The total run time was 28.49min. A C7-C30 standard series (Sigma-Aldrich, Denmark) was used to calculate retention indices (*I*). All data were analysed using the Shimadzu software Lab Solutions, GCMS Solutions version 2.50 SU3. NIST and Wiley 2008 libraries were used in conjunction with the NIST Standard Reference Database for compound identification. Putative compound assignment was made based on a combination of retention index (RI) and mass spectrum similarity, and by comparison to authentic standards for α-guaiene, α-bulnesene, α-copaene, δ-cadinene, α-humulene, and β-caryophyllene.

## Results

### Identification of a novel Vitis vinifera guaiene synthase

**Fig. 1.**
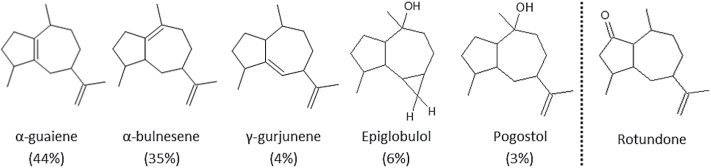
Chemical structures of the five major volatile products of VvGuaS expressed in *N. benthamiana* leaves and analysed via SPME-GCMS. Absolute stereochemistry is not specified. The proportion of each compound detected, as measured by percentage of TIC, is shown below each compound. Note the presence of the common 5,7 bicyclic ring in all structures. The structure of the peppery aroma compound, rotundone, is presented for comparison.

Recent work investigated the global expression of mRNA transcripts in Shiraz grapes and identified a number of potential members of the *TPS-a* subfamily expected to produce sesquiterpenes, and actively transcribed in developing Shiraz berries (Sweetman *et al.*, 2012). The products of these enzymes included a number of monocyclic and bicyclic sesquiterpenes, some of which have previously been identified as products of grape enzymes (Martin *et al.*, 2010). However, one of the TPSs we investigated, encoded by *VvTPS24* (National Center for Biotechnology Information [NCBI] accession XM_002282452), was of particular interest owing to the high proportion of guaiene-type sesquiterpenes produced. The activity of the encoded enzyme was investigated through *Agrobacterium*-mediated transient expression of *VvTPS24* cDNA into *N. benthamiana* leaves, followed by analysis of volatile products produced using solid phase micro-extraction (SPME) GC-MS. Comparison of RIs and mass spectra to those of authentic standards from appropriate GC-MS libraries enabled identification of the major products as α-guaiene (44%) and α-bulnesene (35%, also known as δ-guaiene). A number of minor products were also produced, putatively annotated as epiglobulol, γ-gurjunene, and pogostol, that also exhibit the characteristic guaiene-type 5,7 bicyclic carbon skeleton of the peppery aroma compound rotundone (Fig. 1). Based on this analysis, we named this *VvTPS24* gene product VvGuaS, despite the expectation that it should encode the selinene-producing VvPNSeInt (Martin *et al.*, 2010). GC-MS data for VvGuaS products are presented in the Supplementary data.

### *VvGuaS is a polymorphic variant of the* VvTPS24 *gene*

The array of products detected following expression of this protein was somewhat unexpected given that VvPNSeInt, an enzyme apparently encoded by the *VvTPS24* gene, was previously functionally characterized and found to produce a completely different array of sesquiterpene products. BLAST searches utilizing the nucleic acid sequence of our isolated cDNA, as well as the amino acid sequence of VvGuaS, demonstrated that it was 99.5% identical to a sesquiterpene synthase transcript (NCBI accession XM_002282452). It is on this sequence that the synthetic terpene synthase construct HM807406 was designed, which encoded the selinene synthase VvPNSeInt (Martin *et al.*, 2010). No sequences from the NCBI reference sequence genome, based on the Pinot Noir-derived variety PN40024, matched our variant. However, contig VV78X107636.8 (NCBI accession AM459143), produced as part of the whole genome shotgun sequencing of Pinot Noir (Velasco *et al.*, 2007), contained a predicted protein-encoding region that contained ambiguous nucleotide calls at four out of six positions corresponding to differences between VvGuaS and VvPNSeInt. Specifically, the VvGuaS and VvPNSeInt amino acid sequences differed at positions 403, 405, 414, 431, 499, and 530, and the predicted protein product of contig VV78X107636.8 showed ambiguous residues at all but the first two positions. The presence of these ambiguous base calls at these sites strongly indicates that the *VvTPS24* gene is heterozygous and that alleles encoding both VvGuaS and VvPNSeInt exist in the Pinot Noir genome. Meanwhile, the absence of any sequence corresponding to VvGuaS in the NCBI reference genome suggests that it may have been bred to homozygosity during the development of the PN40024 inbred variety, with only the allele encoding VvPNSeInt remaining.

### Structural comparison of VvGuaS and VvPNSeInt

**Fig. 2.**
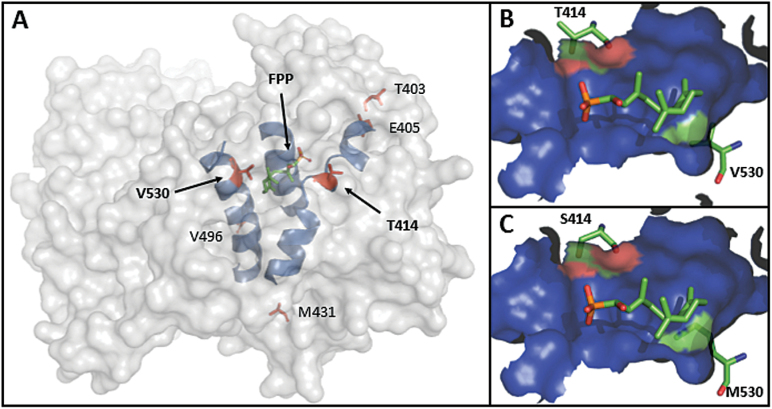
A molecular model of the global VvGuaS protein structure based on the TEAS template. (**A**) A computed enzyme surface is shown as a transparent grey solid, three relevant helices of the secondary structure are visible in blue, and bound FPP, as located in TEAS, is shown centrally located in green (diphosphate group in red). The position of the six amino acid residues that differ between VvPNSeInt and VvGuaS is shown in red and labelled, with T414 and V530 indicated by an arrow. (**B**) Close-up view of part of the internal active site cavity of VvGuaS and (**C**) VvPNSeInt. The molecular surface of the internal cavity is shown predominantly in blue, with the FPP substrate and side-chains of amino acid residues 414 and 530 (VvGuaS numbering used) shown as sticks. The contributions of relevant amino acid residues to the formation of the cavity molecular surface are shown using the equivalent colours of the contributing atoms (carbons in green and oxygen in red).

To investigate the reasons for the dramatic functional differences observed between these two enzymes, a comparison of the structural location of the amino acid difference between VvGuaS and VvPNSeInt was undertaken. Molecular modelling of the two enzymes based on the TEAS template structure revealed that two of the varying amino acid positions were directly located in the active site, proximal to the location of FPP binding and subsequent catalysis, while the other four amino acid differences were located more peripherally (Fig. 2A). The two polymorphisms within the active site of VvGuaS compared to VvPNSeInt corresponded to T414S and V530M substitutions. In both cases, modelling of the electrostatic surface of the active site of VvGuaS demonstrated that the T414 and V530 residues both contribute to the internal binding site of FPP (Fig. 2B, C) and are located on separate alpha-helices that contribute to the formation of the internal cavity comprising the FPP substrate-binding site. The estimated distances from the most proximal atoms of the side-chains of T414 and V530 to FPP in the molecular model of VvGuaS were approximately 4 Å. The remaining four residue differences between VvGuaS and VvPNSeInt, namely T403R, E405D, M431I, and V499F, were located a minimum of 10 Å from the FPP binding site and did not contribute to the molecular surface of the internal cavity (Fig. 2A).

It has previously been demonstrated that amino acid residues located near the site of FPP binding within enzymes of the sesquiterpene synthase family can have profound effects on the metabolites produced, with the observed effects decreasing with decreasing proximity (Greenhagen *et al.*, 2006). To investigate whether the two amino acid residue polymorphisms located at the FPP binding site of VvGuaS could be responsible for the different products generated compared with the previously characterized isoform VvPNSeInt, we carried out site-directed mutagenesis of the *VvTPS24* cDNA to produce versions with either individual (T414S and V530M) or double (T414S/V530M) mutations. *Agrobacterium*-mediated transient transformation of *N. benthamiana* leaves was carried out with these mutated versions of VvGuaS and the volatile metabolites were analysed via SPME GC-MS (Table 1).

**Table 1.** Volatile metabolic products of VvGuaS expressed in *N. benthamiana*

			**TIC%**				
**Compound**	RI	Ref RI^b^	*WT*	T414S	V530M	T414S/V530M	VvPNSeInt^c^
α-copaene^a^	1373	1372–1376	2	3	1	1	-
α*-gurjunene*	1407	1402–1411	1	1	0	0	-
α*-guaiene* ^a^	1437	1428–1437	44	41	24	5	3.5
*Unknown*	1440	n/a	0	0	0	1	-
*γ-gurjunene*	1478	1479	4	3	trace	0	-
*Selina-4(14),11-diene*	1482	1472–1488	0	4	24	40	34
*Epiglobulol*	1488	1463–1497	6	12	2	2	-
α*-selinene*	1492	1478–1497	0	7	16	23	14
*Unknown*	1498	n/a	5	5	4	4	-
α*-bulnesene* ^a^	1506	1500–1515	35	18	12	0	-
*Epi*-α*-selinene*	1517	1518	1	4	8	12	15
*δ-cadinene* ^a^	1524	1519–1530	0	0	3	2	-
*Pogostol*	1653	1637–1656	3	2	0	0	-
*Intermedeol*	1660	1654–1667	0	2	8	9	30

TIC percentages are semi-quantitative because they depend on each compound’s affinity for the SPME fibre. Compound identities reported based on comparison to authentic standards (^a^) or putatively annotated based on mass spectrum similarity (>90% as calculated by Shimadzu GCMS Solution 2.50 software against compounds in NIST08 and Wiley08 libraries) and RI within the range of reported references, except for epi-α-selinene and intermedeol, which are based on RI alone. ^b^ Ranges of reported retention indices for DB5 and similar columns as compiled in the NIST Standard Reference Database 69: *NIST Chemistry WebBook*. ^c^ Products previously reported for VvPNSeInt shown for comparison (Martin *et al.*, 2010).

### Effect of site-directed mutagenesis of selected VvGuaS residues

Compared with wild-type (WT) VvGuaS, the T414S mutant exhibited a relatively minor change in product profile, with the two major volatile metabolites continuing to be α-guaiene (41%) and α-bulnesene (18%). The relatively small decrease in the total proportion of α-bulnesene was replaced by the new products selina-4,11-diene (4%) and α-selinene (7%). These new products were observed at retention times where no trace sesquiterpene ions were visible in the WT VvGuaS enzyme. Additionally, the proportion of epi-α-selinene increased from 1% to 4% of the total ion chromatogram (TIC). Thus, the T414S mutant had 12 identifiable products, compared to nine for the WT enzyme, with each of the new products consisting of sesquiterpenes with a 6,6 bicyclic carbon backbone similar to products of VvPNSeInt (Table 1 and Supplementary data). In the case of VvGuaS-V530M, a more significant change in the range of products was observed, with the overall theme again being a shift towards the product profile reported for VvPNSeInt. The proportions of the 5,7 bicyclic α-guaiene (24%) and α-bulnesene (12%) significantly decreased compared to WT, and substantially higher amounts of the 6,6 bicyclic compounds putatively identified as selina-4,11-diene (24%), α-selinene (16%), and epi-α-selinene (8%) were produced. Pogostol (a hydroxy-guaiene) was no longer present, while a hydroxy-selinene compound, likely intermedeol, now comprised 8% of the TIC. Analysis of the double mutant VvGuaS-T414S/V530M demonstrated that its guaiene synthase activity was almost completely compromised, with only 5% of the volatile products comprising α-guaiene, and α-bulnesene not detectable. Instead, the major products were predominantly the 6,6 bicyclic sesquiterpenes selina-4,11-diene (40%), α-selinene (23%), epi-α-selinene (12%), and a hydroxylated selinene (9%) with the same reported RI as intermedeol.

## Discussion

Following the widespread and relatively rapid domestication and cultivation of grapevine over the past several thousand years (This *et al.*, 2006), there are now up to 10 000 acknowledged varieties of *V. vinifera* (Alleweldt and Dettweiler, 1994). While several hundred of these varieties are commonly used for the production of wine, much remains to be discovered concerning the precise content of aroma-active compounds in most of them. Nevertheless, it is apparent that white wine varieties tend to have a higher relative content of monoterpene compounds, while sesquiterpenes have so far been predominantly found in red wine varieties (Coelho *et al.*, 2006; Vilanova and Sieiro, 2006; Coelho *et al.*, 2007; Kalua and Boss, 2009, 2010; May and Wust, 2012; Matarese *et al.*, 2013; Matarese *et al.*, 2014; Robinson *et al.*, 2014a). While this may, in part, be a result of the localization of sesquiterpene compounds in berry exocarp, and thus heavily influenced by the involvement of berry skin in the winemaking process, studies focusing on berries have shown similar results. This suggests that a complete molecular understanding of the enzymatic pathways involved in sesquiterpene biosynthesis in grapes could provide valuable information regarding the origins of biochemicals present in wine.

The bicyclic sesquiterpene rotundone, first identified in Shiraz wine, is an extraordinarily potent aroma compound with an extremely low detection threshold (Siebert *et al.*, 2008; Wood *et al.*, 2008). Conferring a distinct peppery or spicy note to wine, rotundone is also the first sesquiterpene conclusively shown to have a significant effect on wine character, and has been described as one of the most important aroma compounds in wine (Geffroy *et al.*, 2014). Following its initial identification in Shiraz, a variety that is famous for its peppery character, rotundone has been found in several other cultivars, including Duras, Durif, Graciano, Grüner Veltliner, Schioppettino, and Vespolina (Caputi *et al.*, 2011; Qian and Shellhammer, 2012; Geffroy *et al.*, 2014). Owing to the importance of this compound as a chemical determinant of wine aroma, several studies have investigated the location and timing of rotundone biosynthesis in grapes, and the environmental factors and viticultural practices that may influence final concentrations. Rotundone has been found in exocarp and skin, but is absent from pulp or seeds (Caputi *et al.*, 2011). Absolute levels were shown to increase late in berry ripening and were generally higher in cooler vintages (Caputi *et al.*, 2011; Geffroy *et al.*, 2014). These data add to physiological studies investigating the production of other grape specialized metabolites, whose results have demonstrated that their biosynthesis can be influenced by biotic factors such as insect attack (D’Onofrio *et al.*, 2009), or abiotic factors such as light exposure, crop load, and nitrogen levels (Chapman *et al.*, 2004; des Gachons *et al.*, 2005; Chone *et al.*, 2006).

Given the direct link between rotundone and the peppery aroma of wine, the elucidation of the metabolic pathway of its biosynthesis, and the enzymes responsible for catalytic steps within this pathway, would open up possibilities for investigating grapevine varieties for their potential to produce a pepper aroma. Additionally, techniques such as quantitative mRNA transcript analysis could enable the real-time monitoring of environmental or agronomic factors that affect the expression of the genes responsible. As a sesquiterpene ketone, the most likely biosynthetic pathway of rotundone is via a guaiene intermediate, that is, a bicyclic 5,7 ring carbon skeleton (see Fig. 1), that would be directly generated from FPP by a terpene synthase (Kumeta and Ito, 2010). Indeed, the oxidation of α-guaiene by a fungal laccase has been shown to result in the production of rotundone (Schilling *et al.*, 2013), and rotundone accumulates over time in commercial samples of guaiene that are exposed to the environment (unpublished observation). Recently, Huang *et al.* (2014) demonstrated that rotundone is one of the main products of the auto-oxidation of α-guaiene. Their data suggest that α-guaiene can slowly oxidize to rotundone over a number of weeks at room temperature. This process is dramatically increased at temperatures of 40–50ºC (Huang *et al.*, 2014). If spontaneous conversion of α-guaiene to rotundone plays a role physiologically, this could potentially explain the increased levels of rotundone in grapes that are harvested significantly later than usual (Geffroy *et al.*, 2014; Davies *et al.*, 2015). In contrast, two separate studies have indicated that increased temperature actually correlates with lower rotundone concentrations (Zhang *et al.*, 2013; Scarlett *et al.*, 2014). This suggests that small increases in ambient temperature do not have a significant effect on the rate of any non-enzymatic α-guaiene oxidation that may be occurring.

While it has been shown that α-guaiene can be oxidized to rotundone spontaneously (Huang *et al.*, 2014), there is also the possibility that the final step in rotundone production is carried out enzymatically. The oxidation of sesquiterpene hydrocarbons into more specialized products has been well described in a number of plant species. This would likely be carried out by a member of the cytochrome P450 family. The oxidation of valencene into nootkatone is a specific example of a sesquiterpene hydrocarbon being converted to a ketone in a single step by a cytochrome P450 (Cankar *et al.*, 2011). Numerous other examples exist of sesquiterpene oxidation being carried out by this family of enzymes, predominantly from the diverse CYP71 subfamily (Ro *et al.*, 2006; Pickel *et al.*, 2012). In summary, although there is currently no biochemical evidence that rotundone is enzymatically produced from α-guaiene in grapes, structurally similar compounds have been shown to be produced from the combined action of a terpene synthase and cytochrome P450 in other plants. Therefore, whether it is the non-enzymatic oxidation of α-guaiene that leads to the accumulation of rotundone in some wines, or whether an enzyme exists that can specifically catalyse the formation of rotundone from α-guaiene, is yet to be determined. Sweetman *et al.* (2012) previously reported that transcripts corresponding to *VvTPS24* were present in Shiraz berries during veraison, but were not detected at significant levels at harvest. Although this work reported data from only a single season, if their results prove to be representative of VvGuaS expression, this could suggest that downstream modification of α-guaiene after veraison is crucial in determining rotundone levels.

**Fig. 3.**
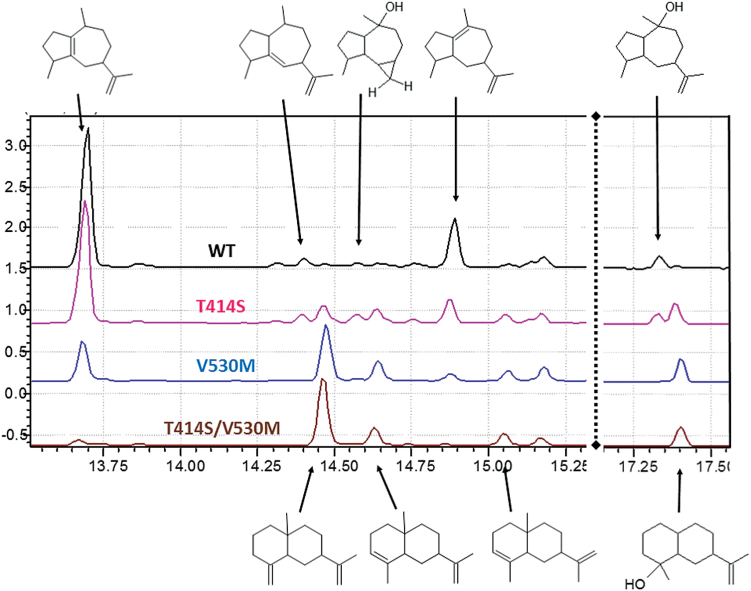
Relevant sections of GC-MS traces, shown as extracted ion chromatograms for marker ion 204, for WT VvGuaS (black), and mutant enzymes T414S (pink), V530M (blue), and T414S/V530M (brown). Major peaks corresponding to specific sesquiterpenes are indicated with black arrows. The vertical dotted line delineates the discontinuous chromatogram. Above are the structures of four major products of WT VvGuaS, comprising (from left to right) α-guaiene, γ-gurjunene, epiglobulol, α-bulnesene, and pogostol. Below are four of the major products of T414S/V530M, and also VvPNSeInt, comprising selina-4,11-diene, α-selinene, epi- α-selinene, and intermedeol.

The findings presented here demonstrate that a newly identified allele of the *VvTPS24* gene encodes VvGuaS, a sesquiterpene synthase whose main product is the rotundone precursor α-guaiene (Fig. 3). We showed that as few as one or two polymorphisms in *VvTPS24* determine whether it will produce α-guaiene (Fig. 3). A previously characterized enzyme, VvPNSeInt, also encoded by *VvTPS24*, was shown to produce selina-4,11-diene (34%) and intermedeol (30%), and only 3.5% α-guaiene (Martin *et al.*, 2010). Both selina-4,11-diene and intermedeol differ structurally from the products of the allele identified in this report, in that they have a 6,6 bicyclic carbon skeleton rather than the 5,7 carbon skeleton of guaiene-type sesquiterpenes. Other than α-guaiene, the only product of VvGuaS that was also reported to be a product of VvPNSeInt was putatively identified as epi-α-selinene, which accounted for 1% of the TIC of VvGuaS compared with 15% for VvPNSeInt (Martin *et al.*, 2010). These data suggest that although the enzymes encoded by the two *VvTPS24* alleles share 99.5% identity at the amino acid level, they clearly have different catalytic functions. Furthermore, analysis of the available sequence data indicates that *VvTPS24* is heterozygotic for the two alleles in the variety Pinot Noir, while the inbred variety PN40024 is likely homozygotic for VvPNSeInt.

**Fig. 4.**
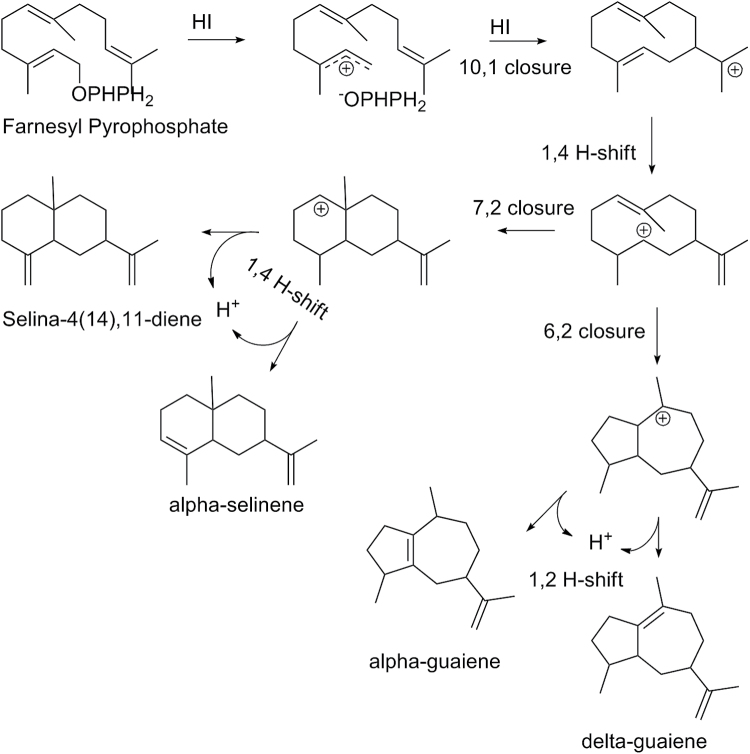
Scheme demonstrating the mechanism of carbocation formation and second ring closure catalysed by VvGuaS (right-hand side) and the T414S/V530M mutant or VvPNSeInt (left-hand side).

In VvGuaS, the double mutation of T414S and V530M, and to a lesser extent the V530M mutation alone, caused a change in the conformation of the active site that resulted in the formation of the second ring favouring a 7,2 closure rather than the 6,2 closure favoured by the WT VvGuaS enzyme (Fig. 4). The products of the double mutant therefore consisted predominantly of sesquiterpenes with a 6,6 bicyclic carbon skeleton, in contrast to WT VvGuaS, which produces a range of guaiene-like sesquiterpenes with a 5,7 bicyclic skeleton (Fig. 3). In other words, the VvGuaS-T414S/V530M double mutant enzyme produced a product profile equivalent to the alternative *VvTPS24* allele, VvPNSeInt. This change in product profile was partially achieved with both of the single mutations T414S and V530M, with the latter having a greater effect. The four major products of the WT VvGuaS enzyme (α-guaiene, α-bulnesene, epiglobulol, and γ-gurjunene) were responsible for 89% of the TIC, while the major four products for the double mutant made up 84% of the TIC, indicating there was no significant decrease in catalytic specificity resulting from the functional change. Similarly, notwithstanding the semi-quantitative nature of SPME GC-MS, no significant differences were observed in the absolute quantities of products produced by the respective enzymes.

Differences in the functionality exhibited by the two highly similar enzymes can potentially be explained by the existence of lone-pair electrons on the sulfur atom of methionine (present in VvPNSeInt), which could be involved in the stabilization of the carbocation intermediate. While the lone-pair on the sulfur is the most likely new interaction partner, differences to the preferred bond closure of the carbocation could be the result of steric changes associated with the relatively minor size differences between the substituted residues (Fig. 2). Although the crystal structure of the WT and mutant enzymes would be required to answer such mechanistic speculation, it has previously been reported that very minor changes to the active site of TPSs can have a significant impact on their products (Greenhagen *et al.*, 2006; O’Maille *et al.*, 2008). In the present study, we used protein-encoding sequences from two alleles of the same gene to direct our mutational analyses and demonstrated that a maximum of two specific residue changes were necessary and sufficient to change the nature of the second ring closure.

In summary, at least two alleles of the *VvTPS24* gene exist in grapevine, both of which encode functional sesquiterpene synthases (VvGuaS and VvPNSeInt). Despite only minor differences in amino acid sequence, we demonstrated that one allele encodes an α-guaiene synthase (VvGuaS), in contrast to the previously investigated version that encodes a selinene synthase (VvPNSeInt). Furthermore, the functional differences between these enzymes can be traced back to two specific polymorphisms in the active site of the protein. The heterozygosity of Pinot Noir for both versions of this enzyme, as evidenced by the existence of contig VV78X107636.8, suggests that it is not only the presence of the α-guaiene-producing allele that is responsible for rotundone accumulation. Nevertheless, the fact that the VvGuaS-producing allele has been discovered in the Shiraz variety, famous for its peppery aroma, could suggest that further investigation into the expression of these two highly similar, but functionally distinct, alleles is warranted. The identification of a grapevine enzyme that produces α-guaiene as its main product is a vital step in determining the molecular basis for rotundone accumulation in grapes, and opens up the possibility for further studies into the factors that influence the peppery aroma of wine.

## Supplementary Data

Supplementary materials are available at *JXB* online.

Supplementary data. Gas chromatogram and mass spectral data for VvGuaS products.

Supplementary Data
