# Supplementary material for: Two key polymorphisms in a newly discovered allele of the Vitis vinifera TPS24 gene are responsible for the production of the rotundone precursor α-guaiene
Source: J Exp Bot. 2015 Nov 17;67(3):799–808. doi: 10.1093/jxb/erv491 (PMC4737073; doi:10.1093/jxb/erv491)
Supplement: Supplementary Data [file supp_67_3_799__index.html]

Two key polymorphisms in a newly discovered allele of the Vitis vinifera TPS24 gene are responsible for the production of the rotundone precursor α-guaiene — Two key polymorphisms in a newly discovered allele of the Vitis vinifera TPS24 gene are responsible for the production of the rotundone precursor α-guaiene — Supplementary Data 

# Two key polymorphisms in a newly discovered allele of the *Vitis vinifera TPS24* gene are responsible for the production of the rotundone precursor α-guaiene

## Supplementary Data

Data files

- Gas\_chromatogram\_and\_mass\_spectral\_data\_for\_VvGuaS\_products.pdf - Supplementary Data
