# Supplementary material for: Two key polymorphisms in a newly discovered allele of the Vitis vinifera TPS24 gene are responsible for the production of the rotundone precursor α-guaiene
Source: J Exp Bot. 2015 Nov 17;67(3):799–808. doi: 10.1093/jxb/erv491 (PMC4737073; doi:10.1093/jxb/erv491)
Supplement: Supplementary Data [file supp_erv491_Gas_chromatogram_and_mass_spectral_data_for_VvGuaS_products.pdf]

# TPS24 double mutant GC-MS chromatogram and MS's

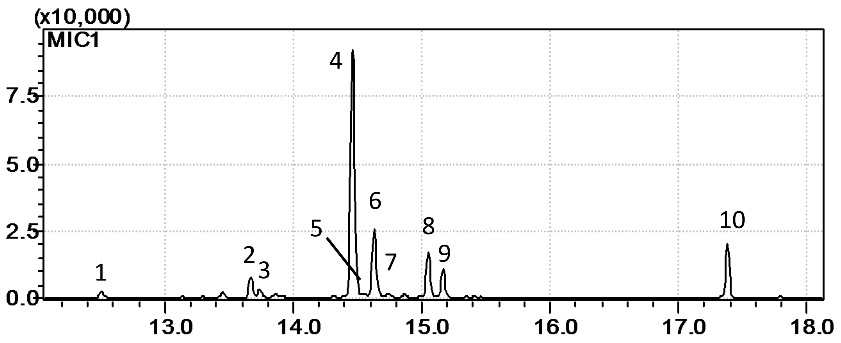


1. Alpha-copaene
2. Alpha-guaiene
3. Unknown
4. Selina-4(14),11-diene
5. Epiglobulol
6. Alpha-selinene
7. Unknown
8. (-)-alpha panasinsene
9. Delta-cadinene
10. Intermedeol?

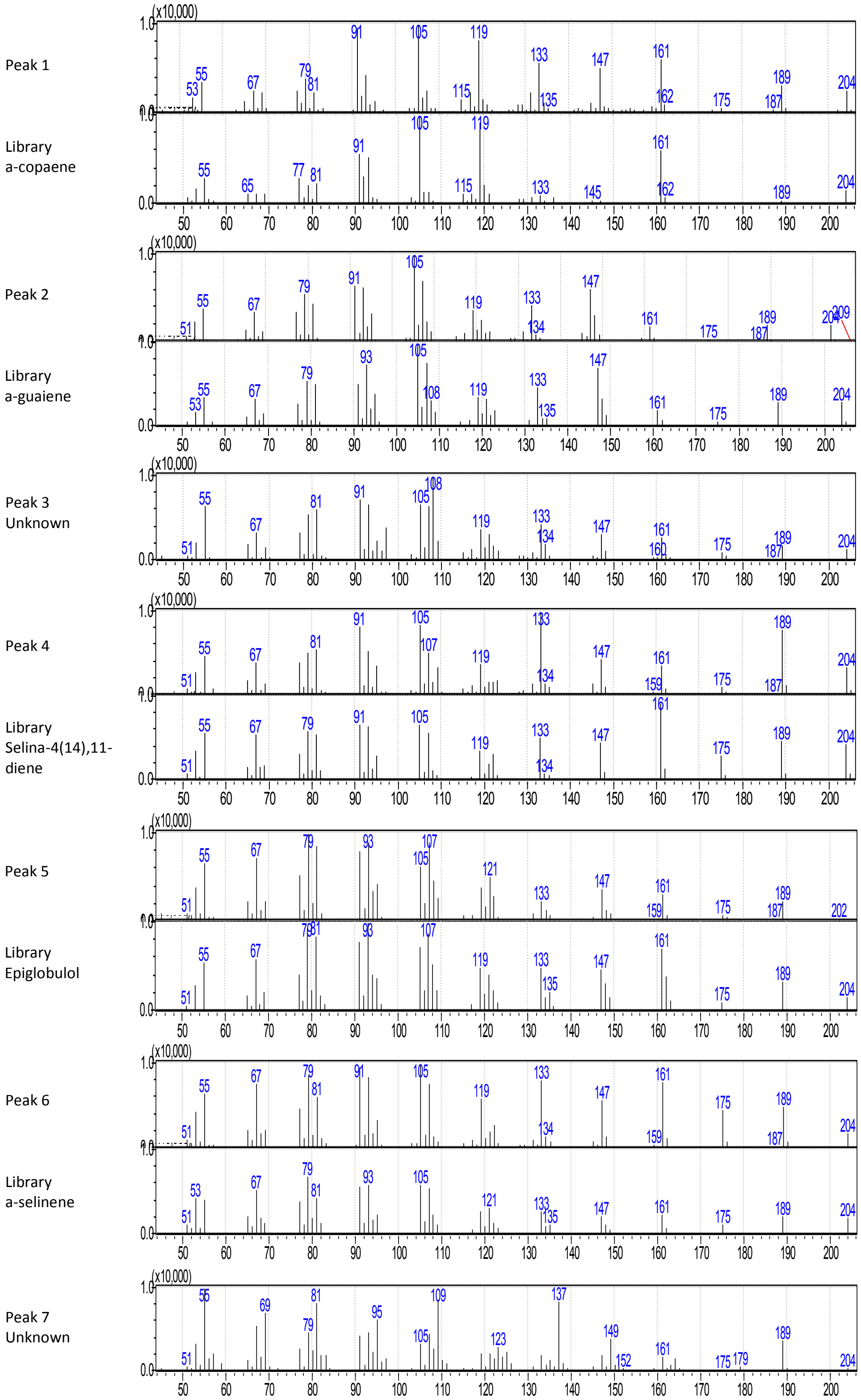

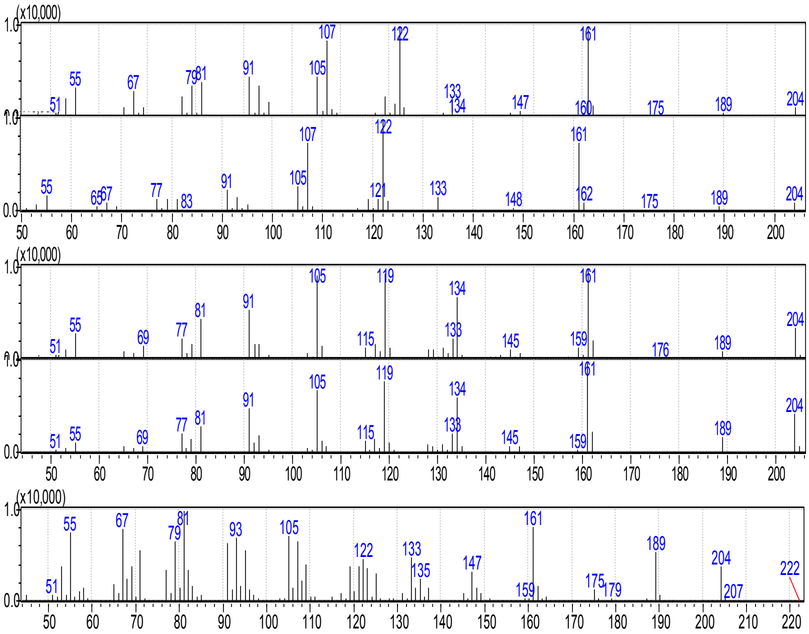


Peak 8

Library
(-)-a-panasinsene

Peak 9

Library
d-cadinene

Peak 10
Intermedeol?

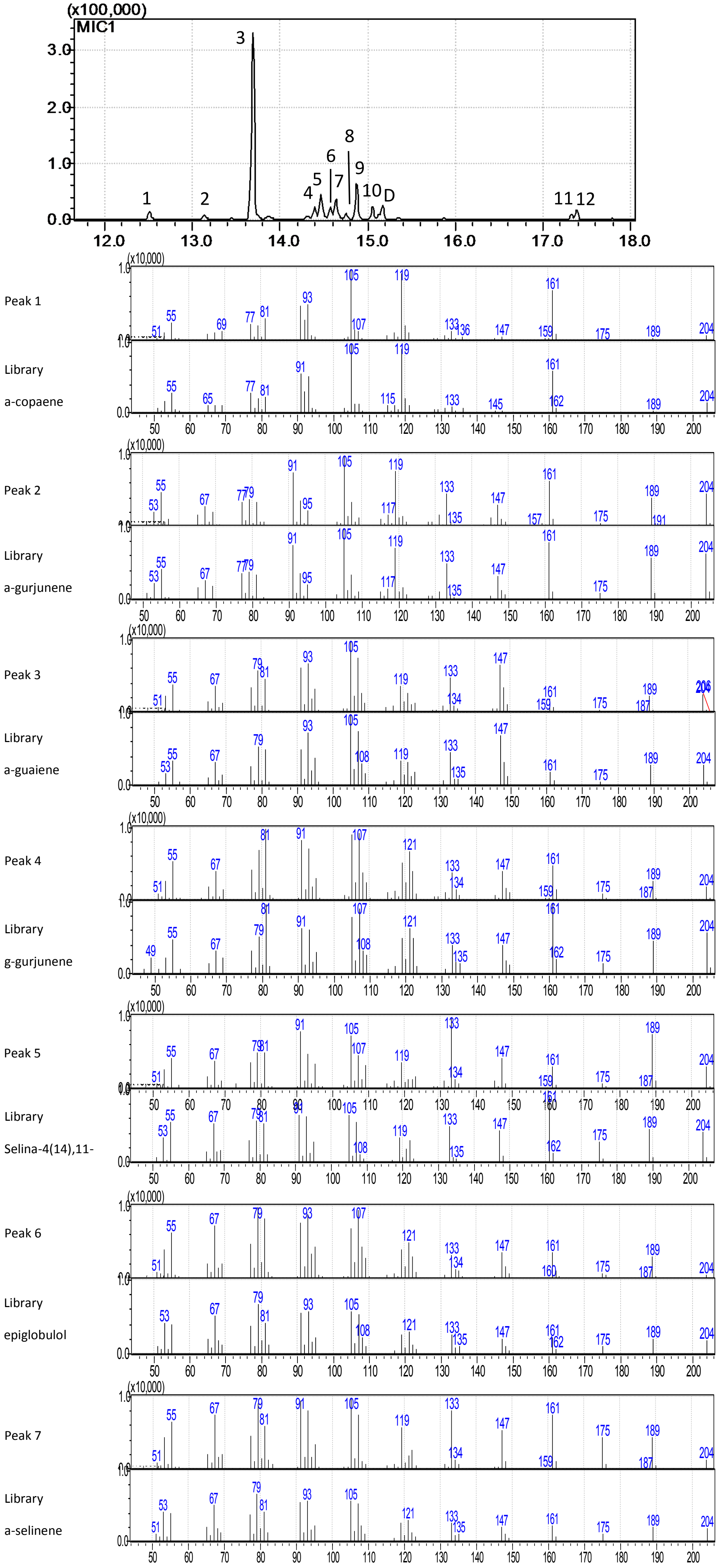


1. Alpha-copaene
2. Alpha-gurjunene
3. Alpha-guaiene
4. Gamma-gurjunene
5. Selina-4(14),11-diene
6. Epiglobulol
7. Alpha-selinene
8. Unknown sesquiterpene

# TPS24 T414S mutant GC-MS chromatogram and MS's

Peak 8

Peak 9

Library

a-bulnesene

Peak 10

Library

Peak 11

Library

pogostol

Peak 12

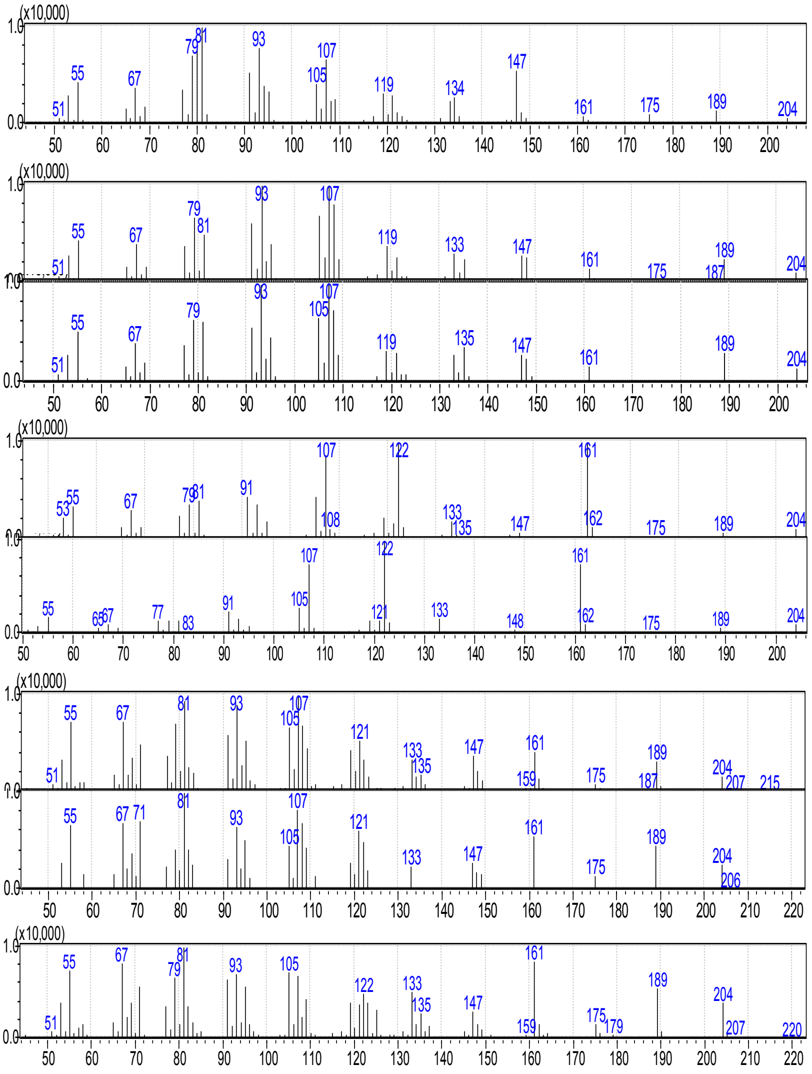

# TPS24 V530M mutant GC-MS chromatogram and MS's

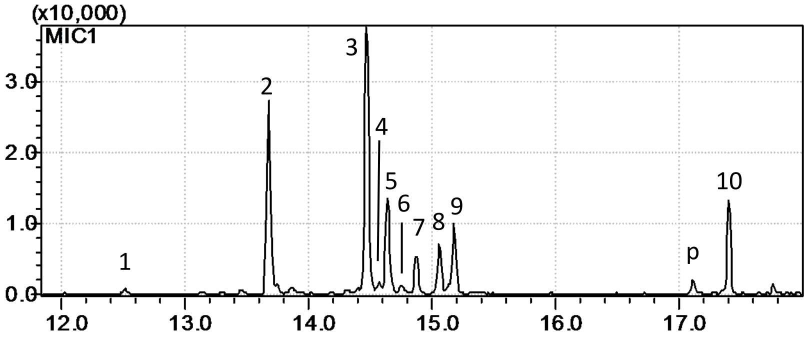


1. Alpha-copaene
2. Alpha-guaiene
3. Selina-4(14),11-diene
4. Epiglobulol
5. Alpha-selinene
6. Unknown sesquiterpene

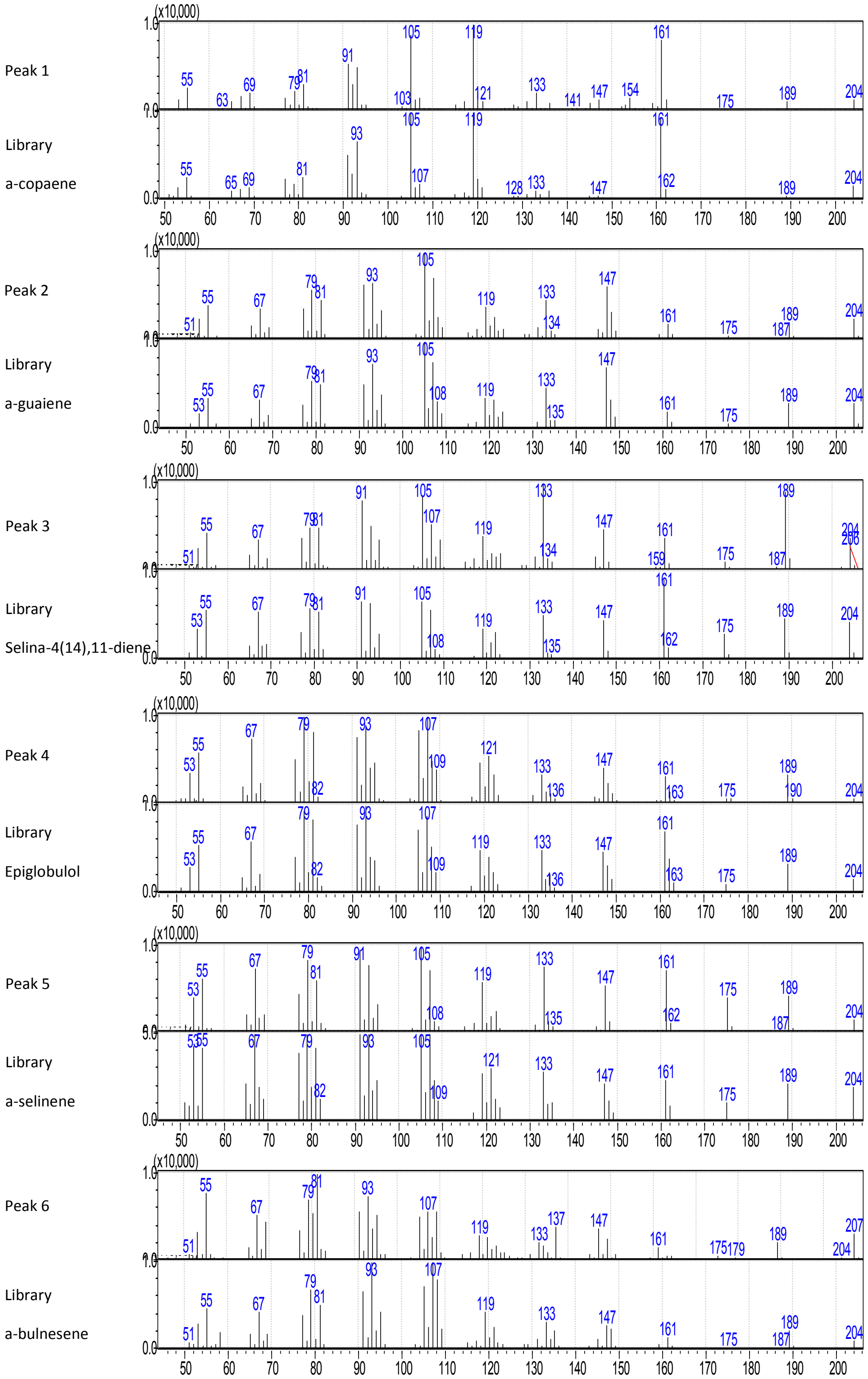

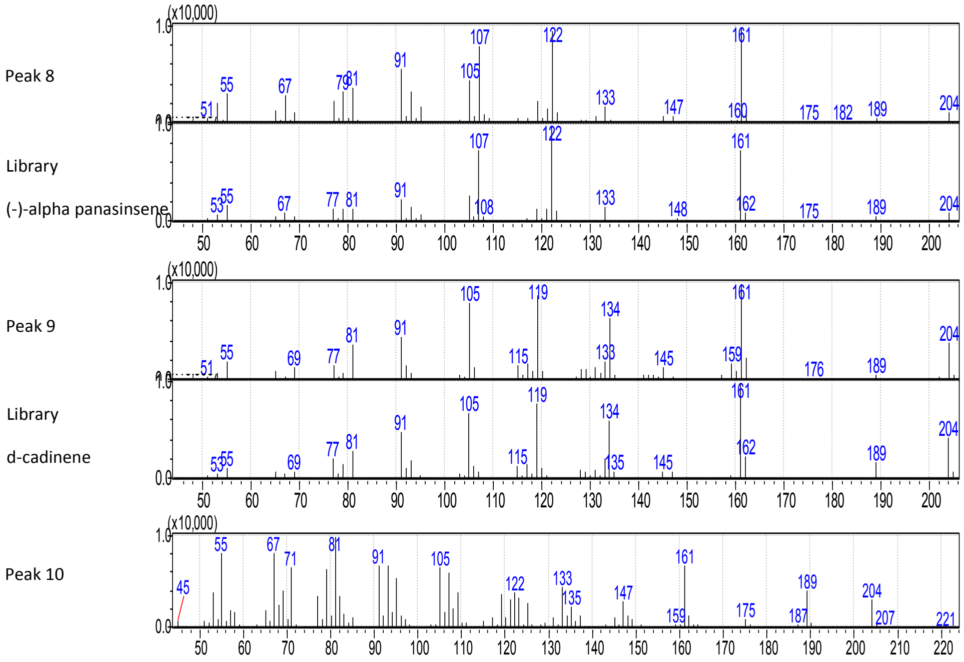

Peak 8
Library
(-)-alpha panasinsene
Peak 9
Library
d-cadinene
Peak 10
(x10,000)

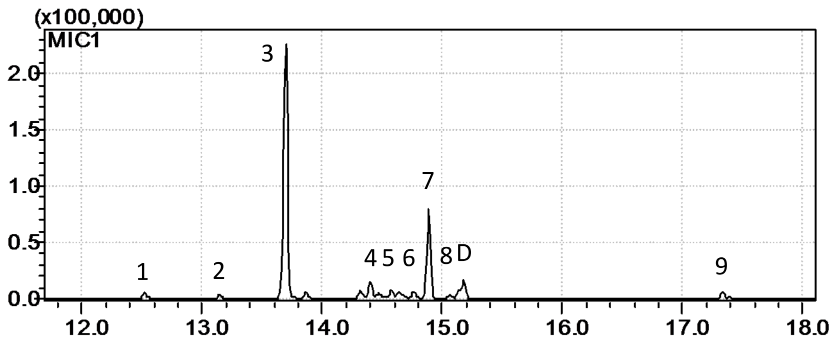


1. Alpha-copaene
2. Alpha-gurjunene
3. Alpha-guaiene
4. Gamma-gurjunene
5. Epiglobulol
6. Unknown sesquiterpene

# TPS24 WT GC-MS chromatogram and MS's

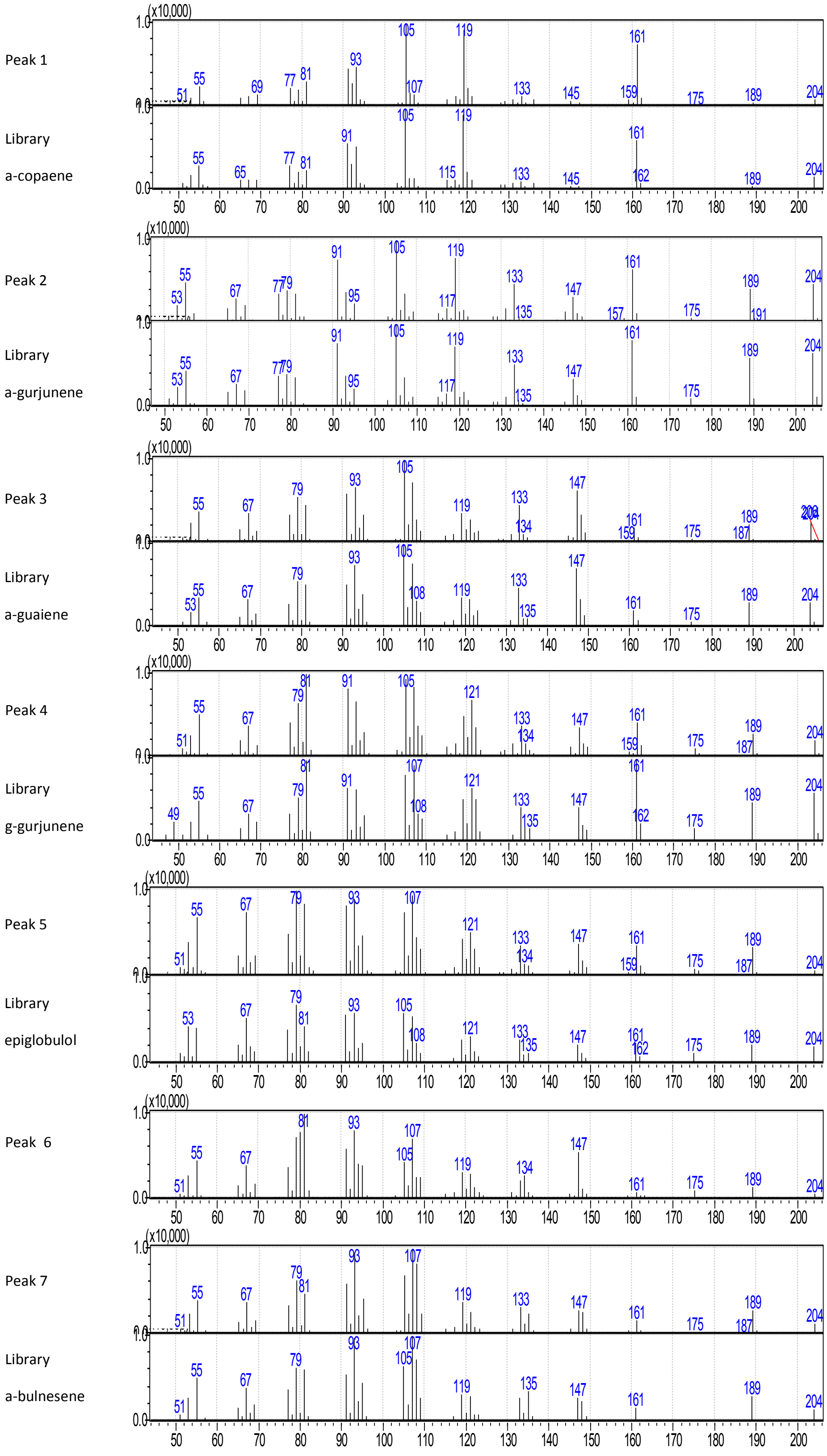

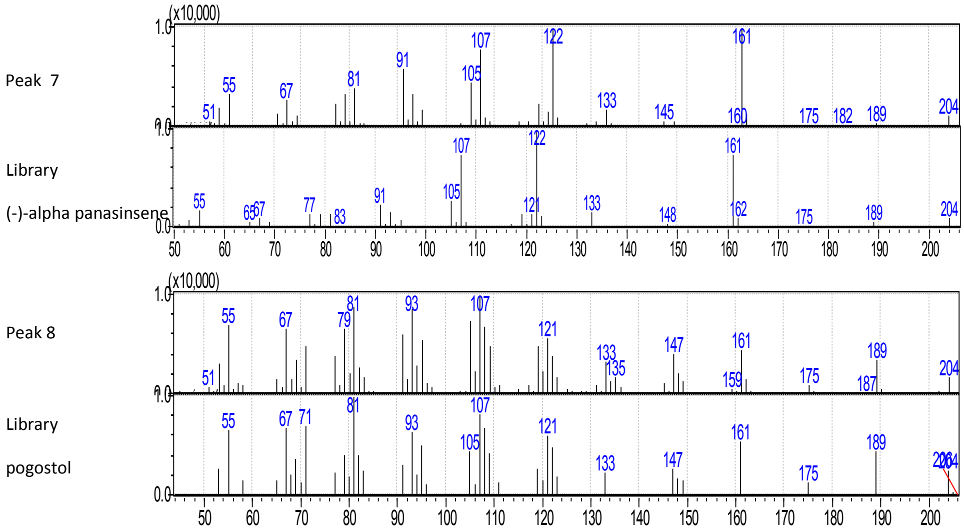
Peak 7
Library
(-)-alpha panasinsene
Peak 8
Library
pogostol
(x10,000)
(x10,000)
